# Functionalized Carbon Nanotubes: Emerging Nanomaterials for Enhanced Cancer Diagnosis and Imaging

**DOI:** 10.3390/molecules30112364

**Published:** 2025-05-29

**Authors:** Anish Prasad Lohani, Mohamed Elosta, Mahmoud Maksoud, Nimer Murshid

**Affiliations:** Biological Sciences, Carnegie Mellon University in Qatar, Educational City, Doha P.O. Box 24866, Qatar

**Keywords:** carbon nanotubes (CNTs), cancer, diagnosis, biosensors, functionalization

## Abstract

Cancer remains a leading global cause of mortality, highlighting the critical need for effective early diagnosis. Despite advancements in treatment, early detection and imaging continue to pose significant challenges. Functionalized carbon nanotubes (CNTs) have emerged as promising nanomaterials due to their unique structural properties and versatile functionalization strategies. This review explores the role of both covalent (e.g., fluorination, hydrogenation, cycloadditions, aryldiazonium salt reduction, organometallic ion attachment, carboxylation, amidation, esterification, and metallic nanoparticle attachments) and non-covalent functionalization methods (e.g., surfactant coating, polymer wrapping, biomolecule attachment, and polymer encapsulation) in enhancing CNT biocompatibility and diagnostic efficiency. Functionalized CNTs are extensively applied in cancer detection through highly sensitive biosensors, including electrochemical, optical, and field-effect transistor-based systems, capable of detecting various cancer biomarkers with exceptional sensitivity. Additionally, they offer significant advantages in cancer imaging modalities such as fluorescence imaging, magnetic resonance imaging (MRI), computed tomography (CT), and ultrasound imaging, improving contrast, resolution, and specificity. This review also discusses the challenges and future directions in the development of CNT-based diagnostic platforms, emphasizing the need for continued research to advance their clinical translation and integration into routine cancer diagnostics.

## 1. Introduction

Cancer is a leading cause of mortality and disability worldwide, characterized by unregulated cell proliferation that can spread and destroy normal tissues [1]. The World Health Organization (WHO) recognizes cancer as a major public health concern. The disease is a significant focus of healthcare efforts [2,3,4].

Research in the field of cancer is crucial, with efforts focused on improved diagnosis [5,6,7], early detection [8], and development of effective therapies [5,9]. Early detection is essential to improving patient outcomes [8,10,11,12,13]. The development of targeted therapies is another critical area of research that seeks to minimize the side effects of cancer treatment and increase recovery rates [14,15,16,17].

Various approaches to cancer treatment are being explored, and nanotechnology has emerged as a promising field for developing novel diagnostic and therapeutic strategies [8,18,19]. Nanomaterials, including carbon nanotubes (CNTs), are being investigated for their potential in cancer therapy. CNTs have arisen as a significant area of study, especially for their roles in both diagnosis and therapy [20,21,22].

CNTs offer several advantages in biomedical applications [23,24]. They have a high surface area, which allows them to carry a large number of therapeutic molecules [21,22,25,26,27]. Additionally, CNTs can be functionalized to enhance their biocompatibility, target cancer cells, and deliver drugs directly to tumor sites [28,29,30]. Furthermore, CNTs’ unique properties make them useful as biosensors and in bioimaging for early cancer detection [31,32,33,34].

CNTs have a unique structure, with diameters in the nanometer range and lengths that can reach micrometers. They can be single-walled (SWCNTs) or multi-walled (MWCNTs), which affects their properties. The carbon atoms in CNTs are arranged in a hexagonal lattice, giving CNTs their unique electronic, thermal, and mechanical characteristics. These properties are influenced by the type of CNT, whether single-walled or multi-walled [20].

The structural functionality of CNTs allows for diverse applications in biomedicine, including drug delivery, tissue engineering, and biosensing. Their ability to be functionalized provides further opportunities for modifying their characteristics to suit various applications [35,36].

This review explores recent progress and advancements in the application of functionalized carbon nanotubes (fCNTs) for cancer diagnosis. It begins by discussing the functionalization strategies of CNTs, focusing on both covalent and noncovalent modifications. The review then examines various approaches for utilizing CNTs in cancer diagnosis, particularly in biosensing and bioimaging, before concluding with insights on future directions.

## 2. Cancer and Carbon Nanotubes: Overview

### 2.1. Cancer

Cancer is one of the most dangerous and concerning health problems globally because of its complexity and high mortality rates. Cancer is characterized by uncontrolled cell growth, the potential to spread to other parts of the body, and resistance to treatment. It can often bypass the immune system, remain undetected until it reaches advanced stages, and return after therapy, making treatment more difficult [2].

**Figure 1:**
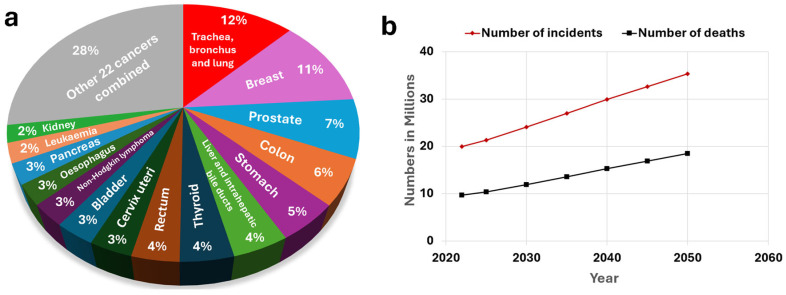
(**a**): Pie chart representation of total global cancer incidences in 2022. (**b**) Number of cancer incidents and cancer-related deaths expected between 2022 and 2050. Data gathered from Refs [2,3].

Another concerning aspect of cancer is its ongoing global burden, which continues to grow at an alarming rate, with both the number of incidents and deaths increasing year by year [2]. In 2022, there were approximately 20 million new cases and nearly 9.74 million cancer-related deaths. Trachea, bronchus, and lung cancer had the highest incidence, accounting for about 12% of global cancer cases, followed by breast cancer at 11%, prostate cancer at 7%, colon cancer at 6%, and stomach cancer at 5% (Figure 1a) [3]. The numbers of incidents and deaths are expected to increase over time, with new cancer cases predicted to hit 35.3 million and about 18.5 million cancer-related deaths by 2050 (Figure 1b) [4]. This escalating burden is expected to strain healthcare systems, deplete critical resources, and impose significant pressure on global economies. As the demand for medical services, research, and treatment rises, it could lead to potential shortages in care and increased inequality in access to treatment [2,3,4].

A study conducted by a group of researchers [37] estimated that the economic cost of cancer from 2020 to 2050 is projected to reach an alarming $25.2 trillion. This estimate includes not only the direct expenses related to cancer treatments but also the broader economic impacts, such as productivity losses and changes in savings and investment patterns resulting from the costs of cancer care.

As cancer rates rise globally, the long-term economic impact will be significant, highlighting the urgent need for early diagnosis and treatment to improve survival, reduce morbidity, lower treatment costs, and decrease the overall economic burden.

### 2.2. Carbon Nanotubes

Due to their exceptional structure (cylindrical hollow nanostructures made from rolled-up sheets of graphene), CNTs exhibit outstanding mechanical properties, including high tensile strength [38], optical properties [39], electronic properties [40], thermal properties [41], electric conductivity [42], and high surface area [43]. These unique characteristics make CNTs ideal for a wide range of applications, including aerospace, composite materials, advanced nanoelectronics, optical sensors, energy storage in batteries and supercapacitors, quantum computing, catalysis, adsorption, and biomedical fields such as drug delivery, medical imaging, and sensing [35].

**Figure 2:**
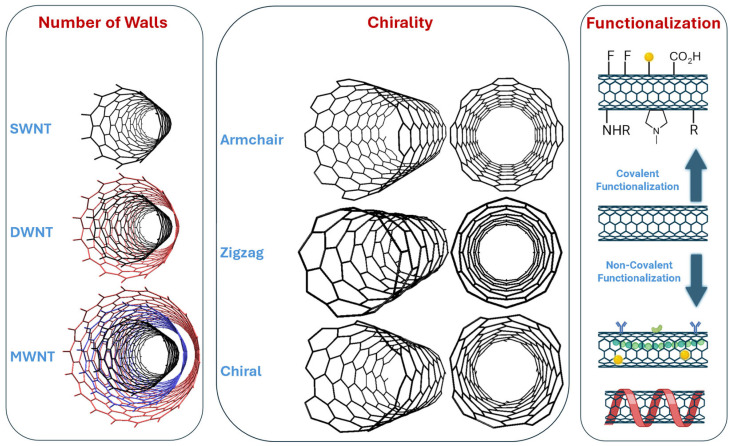
Classification of carbon nanotubes based on number of walls, chirality, and type of functionalization.

As shown in Figure 2, CNTs can be classified into three categories: single-walled nanotubes (SWNTs), double-walled nanotubes (DWNTs), and multi-walled nanotubes (MWNTs). SWNTs consist of a single sheet of graphene rolled into a cylindrical structure. In contrast, DWNTs and MWNTs are composed of two and multiple concentric layers of graphene, respectively, wrapped around one another like a “Russian doll” [44].

The way the graphene sheet is rolled (the chiral vector or orientation) determines the arrangement of the hexagonal graphene network and leads to three types of CNTs: armchair, zigzag, and chiral, each with a distinct geometric structure. The unique structure of each type of nanotube contributes to their distinct mechanical, electrical, and thermal properties. For example, the armchair structure is metallic, whereas zigzag and chiral structures can be metallic or semiconducting [45].

What makes CNTs particularly remarkable is their ability to have their properties tailored, modified, or enhanced through functionalization. As illustrated in Figure 2, functionalization can be categorized into two types: covalent and non-covalent [35]. Covalent functionalization includes methods such as fluorination, carboxylation, 1,3-dipolar cycloadditions, and the attachment of metallic nanoparticles. Non-covalent functionalization, on the other hand, involves techniques like polymer wrapping, polymer encapsulation, protein attachment, and antibody attachment. These modifications can significantly improve the electronic properties of CNTs, enhance their solubility, improve imaging contrast, facilitate targeted therapy and controlled drug release, as well as increase biocompatibility and biosensor performance. Types of CNTs, their functionality, and biomedical applications are discussed and summarized in the following sections.

## 3. Functionalization Strategies for CNTs

**Figure 3:**
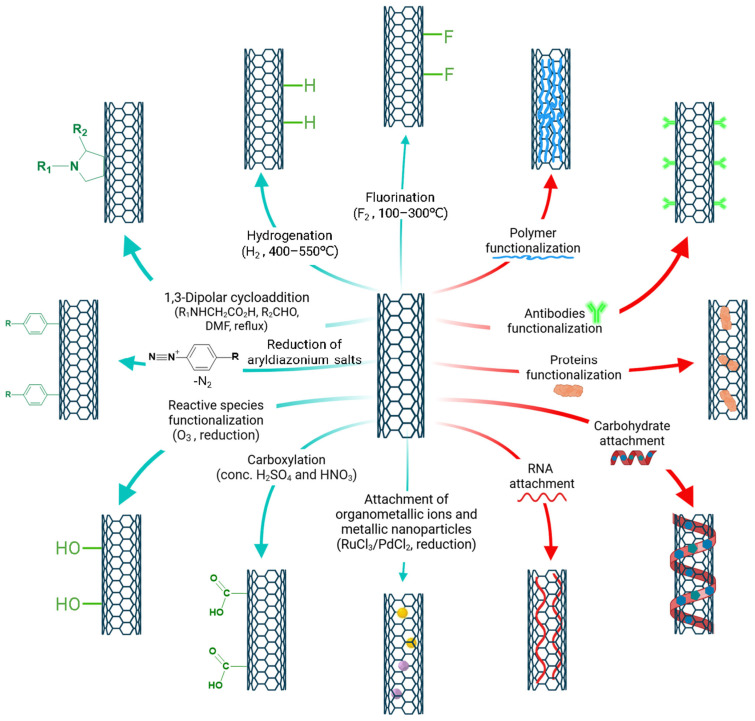
Schematic representation of CNT functionalization strategies: covalent (blue arrows) and noncovalent (red arrows) approaches. Created with BioRender.com.

Section 3 focuses on the diverse functionalization strategies for CNTs, differentiating between covalent and noncovalent modification methods. These critical processes are used to tailor CNT properties, enhancing their performance for specific applications, including improvements in electronic properties, solubility, imaging, and drug delivery. These strategies will be discussed in the following subsections, with their details and comparison summarized in Figure 3.

Functionalized CNTs have emerged as a pivotal tool in the realm of cancer diagnostics and treatment, showcasing their versatility across various cancer types. Recent studies have highlighted their application in targeted drug delivery systems, significantly enhancing the efficacy of chemotherapeutic agents. For instance, dual-targeting multi-walled carbon nanotubes have been utilized to improve the delivery of neratinib in breast cancer, demonstrating superior cytotoxic effects compared to conventional methods [46]. Gelatin-functionalized CNTs loaded with cisplatin have shown promising results in anti-cancer therapy, indicating that functionalization is crucial for maximizing therapeutic outcomes [47]. In the context of photothermal therapy, hyaluronic acid-modified CNTs have been effective in promoting apoptosis in nasopharyngeal carcinoma cells, showcasing their potential in localized cancer treatment [48]. The integration of CNTs in combination therapies, such as those involving radiation sensitizers, has been explored to enhance the effectiveness of radiotherapy in breast cancer [49]. Overall, the application of faunctionalized carbon nanotubes spans various cancer types, including breast cancer and nasopharyngeal carcinoma, underscoring their role as a transformative approach in cancer diagnostics and treatment [48,49].

### 3.1. Covalent Functionalization

The covalent functionalization strategies of CNTs discussed in this subsection are summarized in Figure 3 and indicated by the green arrows.

#### 3.1.1. Fluorination

The fluorination of CNTs has gathered significant attention due to its potential to modify the electronic and chemical properties of these nanomaterials. Fluorination typically introduces C–F bonds, which can alter the electronic structure and enhance the reactivity of CNTs. For instance, it has been observed that fluorinated SWCNTs (fSWCNTs) maintain their structural integrity while exhibiting dispersed fluorine patterns, characterized by a low fluorination degree and a reduced concentration of edge C–F groups [50]. This unique behavior is attributed to the high crystallinity of the carbon network, which allows for selective fluorination without compromising the overall tube structure. Furthermore, the introduction of fluorine can significantly increase the solubility of CNTs in polar organic solvents, enhancing their compatibility with various chemical environments due to dipole interactions [51].

Moreover, the fluorination process can lead to the formation of both covalent and semi-ionic C–F bonds, which are crucial for applications in energy storage and catalysis. For example, studies have shown that fluorination can enhance the electrochemical properties of CNTs, making them suitable for use in lithium batteries and as electrocatalysts [52,53]. The presence of fluorine modifies the electronic properties and influences the hydrophobicity of the CNT surface, which can be beneficial in applications requiring water stability and proton conductivity [54]. Additionally, the interaction between fluorinated CNTs and other materials, such as fluorosumanenes, has been explored to further enhance carrier doping and improve the performance of electronic devices [55]. Overall, the fluorination of carbon nanotubes represents a promising avenue for advancing the functionality of these versatile nanomaterials across various applications. Wang et al. [56] added silver nanoparticles (AgNps) to construct hydro-soluble fluorinated carbon fiber oxide (FCO)/Ag composite, which proved to be a highly effective targeted nanocarrier for photothermal therapy and also demonstrated antibacterial activity. FCO/Ag acts as a fluorescence quencher for doxorubicin, enabling visual monitoring of drug adsorption processes through a “turn-off” fluorescence mechanism. Fluorinated graphene and CNTs have been utilized in a surface plasmon resonance (SPR) sensor proposed by Uniyal et al. for the detection of SARS-CoV-2 viral particles [57].

#### 3.1.2. Hydrogenation

The hydrogenation of CNTs is a significant area of research due to its potential to enhance the chemical reactivity and electronic properties of these nanomaterials. Hydrogenation can introduce hydrogen atoms into the CNT structure, leading to the formation of C–H bonds, which can modify the electronic band structure and improve the material’s catalytic properties. For instance, studies have shown that hydrogenation can enhance the adsorption capacity of CNTs for various gases, including hydrogen itself, thereby making them suitable candidates for applications in hydrogen storage and fuel cells [58]. Additionally, the hydrogenation process can facilitate the growth of nitrogen-doped carbon nanotubes (NCNTs) on substrates like foamed nickel, which can further improve their performance in catalytic reactions. Research indicates that the curvature and electronic properties of CNTs can be optimized through controlled hydrogenation, leading to improved catalytic performance [59]. The interaction between hydrogenated CNTs and metal nanoparticles, such as platinum, has also been explored, revealing that hydrogenation can enhance the bonding strength between the metal and the CNT, thus improving the overall efficiency of catalytic processes [60]. The synthesis of CNTs through hydrogen-rich environments, such as during pyrolysis processes, has been shown to yield high-quality CNTs with desirable properties for various applications, including energy conversion and storage [61].

#### 3.1.3. 1,3-Dipolar Cycloadditions

The 1,3-dipolar cycloaddition (1,3-DC) of carbon nanotubes (CNTs) has emerged as a promising strategy for functionalizing these nanomaterials, enhancing their chemical reactivity, and expanding their application potential. This reaction typically involves the interaction of a dipolar species, such as azomethine ylides, with a suitable dipolarophile, leading to the formation of five-membered heterocycles. Recent studies have demonstrated that encapsulating 1,3-dipolar cycloaddition reactions within CNTs can significantly influence the regioselectivity and stereochemistry of the products, offering a unique environment that can stabilize transition states and intermediates [62]. Theoretical investigations have provided insights into the mechanisms underlying these reactions, revealing that the confinement within CNTs can enhance the reactivity of the dipoles and facilitate the formation of desired cyclic adducts [62]. Furthermore, the use of metal-catalyzed approaches, such as copper-catalyzed azide–alkyne cycloaddition (CuAAC), has been shown to be particularly effective in promoting 1,3-DC reactions [63]. The involvement of CNTs has led to the synthesis of functionalized derivatives that exhibit improved properties for applications in drug delivery and materials science.

Recent research indicates that the functionalization of MWCNTs using the 1,3-dipolar cycloaddition approach with azomethine ylides leads to the development of hybrid materials with improved photodynamic and photothermal properties, making them suitable for treating various cancers, including melanoma and breast cancer [64]. The regioselectivity of these cycloadditions, modulated by the confinement effects within the nanotubes, allows for the precise synthesis of triazole derivatives, which can be utilized as drug delivery vehicles or imaging agents in cancer diagnostics [62,65]. The incorporation of therapeutic agents into these functionalized CNTs has shown promise in enhancing the efficacy of chemotherapy, particularly in colorectal cancer, where targeted delivery can significantly reduce systemic toxicity [19,66]. The versatility of 1,3-dipolar cycloaddition in modifying CNTs underscores its potential in advancing cancer treatment modalities, including targeted therapies and diagnostics across a spectrum of cancer types, such as skin cancer and nasopharyngeal carcinoma [67,68].

#### 3.1.4. Reduction of Aryldiazonium Salts

The reduction of aryldiazonium salts enables the generation of aryl radicals that can participate in various coupling reactions. Aryldiazonium salts, typically formed from aniline derivatives, can be reduced using a variety of methods, including electrochemical reduction and chemical reduction with reducing agents such as zinc or sodium sulfite. Recent advancements have highlighted the utility of transition metal catalysts in facilitating these reductions, which can enhance the selectivity and efficiency of the process [69]. For example, studies have shown that the use of palladium or copper catalysts can significantly improve the yields of aryl radicals from aryldiazonium salts, allowing for more efficient coupling with nucleophiles or other electrophiles [70]. The reduction of aryldiazonium salts can also be performed under mild conditions, making it a versatile method for synthesizing functionalized aromatic compounds ]. The generated aryl radicals can then be utilized in various reactions, including the formation of C–C and C–N bonds, thereby expanding the synthetic utility of aryldiazonium salts in the development of complex organic molecules [71].

Recent studies demonstrate that the electrochemical reduction of aryldiazonium salts can effectively modify the surface of CNTs, enabling the attachment of various biomolecules (nucleic acids, proteins, carbohydrates, etc.), thereby enhancing their targeting capabilities for cancer cells. For instance, functionalized CNTs have been utilized for the targeted delivery of chemotherapeutic agents in breast cancer, improving drug efficacy while minimizing side effects [72]. Additionally, the incorporation of these modified CNTs in biosensors has facilitated the detection of cancer biomarkers, allowing for early diagnosis of cancers such as colorectal and gastric cancer [24,73]. The ability to create controlled monolayer films through the reduction of diazonium salts further enhances the specificity and sensitivity of these biosensors, making them invaluable in the clinical setting [74]. The functionalization of CNTs via aryldiazonium salt reduction not only aids in the targeted treatment of cancers, including breast and colorectal cancer, but also plays an essential role in the development of advanced diagnostic tools that can detect cancer at earlier stages [24].

#### 3.1.5. Reactive Species Functionalization

This process often includes adding functional groups onto the CNT surface through various chemical reactions, including the use of aryldiazonium salts, which can covalently bond to the CNTs, thereby modifying their electronic and chemical properties [75]. The functionalization can also be achieved through hydrogenation, where molecular hydrogen reacts with the CNTs, leading to the formation of stable C–H bonds that can further facilitate additional chemical modifications [76,77]. The incorporation of transition metals or metal oxides into CNTs has been shown to improve their catalytic properties, particularly in hydrogen storage applications, where the presence of these metals enhances the adsorption capacity of hydrogen [78].

The use of reactive oxygen species generated from CNTs has been investigated for their ability to induce oxidative stress in biological systems, which can be harnessed for therapeutic applications [79]. The functionalization of CNTs with various reactive species not only improves their compatibility with different solvents and matrices but also enhances their performance in potential applications such as sensors, drug delivery systems, and catalysis [80].

#### 3.1.6. Organometallic Ions

Organometallic ions play a crucial role in various chemical processes, particularly in catalysis and functionalization reactions involving carbon nanostructures such as CNTs and graphene. These ions can facilitate the formation of covalent bonds between organometallic species and carbon nanostructures, enhancing their reactivity and enabling the development of novel materials with tailored properties. For instance, the functionalization of CNTs through 1,3-dipolar cycloaddition reactions has been shown to effectively incorporate organometallic ions, leading to improved electronic and catalytic properties [81].

Moreover, the use of organometallic ions in conjunction with transition metals has been explored to enhance the selectivity and efficiency of cycloaddition reactions involving CNTs. Studies have demonstrated that the presence of these ions can stabilize transition states and intermediates, thereby promoting desired reaction pathways and improving product yields [82]. The confinement of reactive species within the unique geometry of CNTs can lead to distinct regioselectivity in functionalization reactions, further highlighting the importance of organometallic ions in these processes [65].

Recent studies have demonstrated that the functionalization of CNTs with organometallic ions, such as ruthenium and palladium, enhances their efficacy in drug delivery systems, particularly for breast and prostate cancers [83,84]. These organometallic-functionalized CNTs can effectively carry chemotherapeutic agents, allowing for controlled release and improved targeting of cancer cells, thus minimizing side effects associated with traditional chemotherapy [19]. Furthermore, the integration of organometallic ions into CNTs has been explored for their role in enhancing imaging techniques, such as magnetic resonance imaging (MRI) and computed tomography (CT), facilitating the early detection of cancers like colorectal and lung cancer [19,85]. The ability of these modified CNTs to act as biosensors for detecting cancer biomarkers also highlights their dual role in both diagnostics and treatment, making them invaluable in the fight against various cancer types [19,86].

#### 3.1.7. Carboxylation and Further Derivation

Recent findings indicate that carboxylation can be effectively achieved using aryldiazonium salts as reactive intermediates, facilitating the introduction of carboxylic acid functional groups onto the CNT surface [87]. The functionalization of CNTs through carboxylation can lead to the formation of various derivatives that are useful in drug delivery systems, sensors, and catalysis. The introduction of carboxyl groups can also facilitate subsequent reactions, such as amidation or esterification, thereby enabling the synthesis of more complex organic structures [88]. The ability to control the degree of carboxylation and the nature of the substituents on the CNTs allows for the fine-tuning of their properties, making them suitable for specific applications in materials science and nanotechnology [89].

Recent studies have demonstrated that carboxylated MWCNTs (c-MWCNTs) can effectively serve as carriers for targeted drug delivery systems, significantly improving the therapeutic efficacy of chemotherapeutic agents in various cancers, including breast and colorectal cancer [46]. For instance, c-MWCNTs have proven to boost the cytotoxic effects of cisplatin against breast cancer cells, facilitating preferential drug delivery to cancerous tissues [46]. The functionalization of CNTs with carboxyl groups has been utilized in the development of electrochemical immunosensors for detecting cancer biomarkers, such as nuclear matrix protein 22, which is relevant for bladder cancer diagnostics [90]. The ability of SWCNTs to form stable dispersions in aqueous solutions also enhances their application in biosensing technologies, allowing for the early detection of cancers like gastric and lung cancer [75]. Studies have been done on utilizing carboxylated MWCNTs (MWCNTs-COOH) as a drug delivery system for enhanced ovarian cancer treatment [91]. A system was designed where MWCNTs-COOH was coupled with folic acid to target ovarian tumors and loaded with curcumin (CUR). The carboxyl functional group (-COOH) of MWCNTs-COOH facilitates conjugation with targeting ligands like folic acid, improves drug loading, and enhances cellular absorption. This approach aims to overcome the drawbacks of conventional chemotherapy by enabling more specific and effective drug delivery [91]. The carboxylation of CNTs not only improves their utility in drug delivery systems but also plays a crucial role in advancing diagnostic tools for various cancer types, thereby contributing to more effective cancer management strategies.

Further functionalization of carbonylated CNTs has also been explored, including amidation and esterification of carboxylic groups, which are essential transformations in organic synthesis. Recent advancements in these reactions have focused on improving efficiency and selectivity through innovative catalytic and electrochemical systems [92,93]. Recent research indicates that the functionalization of CNTs through amidation can improve their biocompatibility and facilitate the targeted delivery of chemotherapeutic agents, particularly in breast and colorectal cancers [47,66]. For example, gelatin-functionalized CNTs, achieved through esterification, have been utilized to create pH-responsive drug delivery systems that enhance the release of cisplatin—a common chemotherapeutic agent—specifically in tumor environments [47]. The incorporation of organometallic compounds has been explored to enhance the efficacy of photothermal therapy, where modified CNTs can selectively target and ablate cancer cells without harming healthy tissues [94]. Additionally, the functionalization of CNTs with carboxylic groups allows for the development of advanced biosensors capable of detecting cancer biomarkers, facilitating early diagnosis of cancers such as gastric and lung cancer [73,90].

In addition to amidation, etherification of CNTs has emerged as a significant strategy in enhancing cancer diagnostics and treatment, primarily through the functionalization of CNTs to improve their biocompatibility and drug delivery capabilities. Recent studies have demonstrated that etherified CNTs can effectively serve as carriers for anticancer drugs, allowing for targeted delivery systems that enhance the therapeutic efficacy of agents such as cisplatin in breast and colorectal cancers [24,47]. For instance, the incorporation of ether linkages facilitates the attachment of various biomolecules, which can improve the selectivity of drug delivery to cancer cells while minimizing off-target effects [95]. Additionally, etherified CNTs have been utilized in the development of biosensors for detecting cancer biomarkers, thereby aiding in the early diagnosis of cancers such as prostate and gastric cancer. The ability of these modified CNTs to form stable dispersions in biological fluids further enhances their application in both therapeutic and diagnostic contexts, making them versatile tools in the fight against various cancer types.

Ionic functionalization of carboxylic groups has emerged as a significant area of research, particularly in enhancing the properties of various materials for applications in energy storage and separation technologies [96,97,98]. The ionic functionalization of carboxylic groups not only improves the performance of materials in various applications but also opens new avenues for the design of advanced functional materials such as CNTs.

#### 3.1.8. Attachment of Metallic Nanoparticles

The attachment of metallic nanoparticles to various substrates, particularly to CNTs, has garnered significant attention due to their potential applications in fields such as catalysis, sensing, and biomedical technologies. Recent studies have highlighted innovative methods for the synthesis and functionalization of metallic nanoparticles, particularly through green synthesis approaches that utilize plant extracts and biological systems. For instance, the use of plant-derived extract as reducing and stabilizing agents has been shown to facilitate the eco-friendly synthesis of nanoparticles that, when combined with CNTs, exhibit enhanced properties for various applications such as photothermal cancer therapy [94]. Additionally, research has demonstrated that the coordination-assisted surface functionalization method can effectively graft organic ligands onto substrates like MXene, promoting the uniform growth of ultrafine metal nanoparticles [97]. This method not only enhances the stability of the nanoparticles but also allows for precise control over their size and distribution, which is crucial for optimizing their performance in applications such as drug delivery and catalysis [97]. Overall, the attachment and functionalization of metallic nanoparticles through these innovative approaches represent a significant advancement in nanotechnology, paving the way for the development of next-generation materials with tailored functionalities. For instance, gold nanoparticles have been shown to act as radiosensitizers, enhancing the effectiveness of radiotherapy by increasing the localized radiation dose delivered to tumor cells while minimizing damage to surrounding healthy tissues [49]. The integration of metallic nanoparticles with CNTs not only enhances drug delivery systems but also plays a crucial role in the advancement of diagnostic tools, making it a promising strategy in the fight against various cancer types.

### 3.2. Non-Covalent Functionalization

The non-covalent functionalization strategies of CNTs discussed in this subsection are summarized in Figure 3 and indicated by the red arrows.

#### 3.2.1. Polymer Functionalized CNTs

CNTs can be non-covalently functionalized by polymers through wrapping, encapsulation, or absorption approaches. Polymer wrapping of CNTs has emerged as a promising approach to enhance the stability, functionality, and application potential of these nanomaterials. Recent studies have demonstrated that encapsulating CNTs within polymer matrices can significantly improve their mechanical and thermal properties, making them suitable for various applications, including electronics and energy storage. For instance, a study on PVC/CNT electrospun composites revealed that while some CNTs were not fully encapsulated due to their non-uniformity, the overall morphology and thermal behavior of the composites were positively influenced by the presence of CNTs, suggesting that proper alignment and dispersion are crucial for maximizing performance [99]. The encapsulation of CNTs in N,P-doped graphene has been explored as a method to create high-efficiency trifunctional electrocatalysts, where the polymer wrapping not only stabilizes the CNTs but also enhances their electrochemical activities for applications in flexible batteries and water splitting [100]. Furthermore, the development of dye-encapsulated CNTs has shown potential in photocatalytic applications, indicating that polymer wrapping can facilitate the integration of functional materials for advanced technological uses [101].

Recent studies have shown that wrapping CNTs with conjugated polymers can significantly enhance their drug delivery capabilities, allowing for the effective transport of chemotherapeutic agents such as doxorubicin and cisplatin to cancer cells, particularly in breast and lung cancers [102]. For instance, the polymer wrapping not only stabilizes the CNTs in biological environments but also facilitates the controlled release of drugs in response to specific stimuli, such as pH changes in tumor microenvironments [103]. Additionally, polymer-wrapped CNTs have been utilized in the development of biosensors for detecting cancer biomarkers, enabling early diagnosis of cancers such as colorectal and prostate cancer [104]. The ability of these modified CNTs to form stable dispersions enhances their application in both therapeutic and diagnostic contexts, making them versatile tools in the fight against various cancer types [105].

Moreover, the incorporation of surfactants into polymer matrices has been explored to improve the mechanical and thermal properties of the resulting materials. For example, the addition of dodecyl surfactants to polyamide-6 nanofibers has been found to induce crystalline modifications, transforming the crystalline phase from γ to α-form, which enhances the material’s overall performance including wettability of surfaces, facilitating better fluid movement in microfluidic devices, which is critical for biomedical applications [106,107].

Polymer encapsulation of CNTs has appeared as a vital strategy for enhancing the properties and functionalities of these nanomaterials in various applications. Recent studies have demonstrated that the encapsulation of CNTs within polymers can significantly improve their mechanical, thermal, and electrical properties. For instance, the use of conjugated polymers has been shown to spontaneously wrap around semiconducting SWCNTs, effectively preventing charge dissipation and enhancing the performance of optoelectronic devices [108]. This encapsulation not only stabilizes the CNTs but also facilitates their integration into polymer matrices, leading to composites with improved conductivity and mechanical strength. Additionally, research has indicated that the encapsulation of CNTs in polymers can enhance their compatibility with various solvents and matrices, which is crucial for applications in drug delivery and biosensing [109]. Furthermore, the incorporation of CNTs into polymer systems has been explored for developing advanced materials with multifunctional properties, such as shape memory and electromagnetic shielding [109,110].

Polymer absorption into CNTs enhances the mechanical, thermal, and electrical properties of composite materials. The absorption of sulfur-containing polymers onto CNTs has been demonstrated to create composite cathode materials for lithium–sulfur batteries, resulting in enhanced electrochemical performance due to the effective interaction between the polymer and the CNTs [111]. Additionally, the optimization of polyamide-based nanocomposites with CNTs has highlighted the role of polymer absorption in increasing the impact strength and elastic modulus of the resulting materials, showcasing the importance of uniform dispersion and interfacial bonding for maximizing mechanical properties [112]. Furthermore, the development of biodegradable polymer-CNT composites has been explored for their potential in electromagnetic shielding applications, where the absorption of polymers contributes to the overall performance of the composite [113].

#### 3.2.2. Antibodies Functionalized CNTs

The attachment of antibodies to CNTs has emerged as a promising strategy in cancer diagnostics and treatment, leveraging the unique properties of CNTs to enhance the specificity and efficacy of therapeutic agents. CNTs functionalized with antibodies can selectively target cancer cell markers, thereby facilitating the precise delivery of chemotherapeutic agents. For instance, a study highlighted the use of antibody-conjugated CNTs to detect E-cadherin and keratin-19, which are highly expressed in breast cancer cells, showcasing the potential of these nanoprobes for targeted diagnostics [105]. This approach improves the accuracy of cancer detection and allows for real-time monitoring of treatment responses, making it a valuable tool in personalized medicine.

In addition to diagnostics, antibody attachment to CNTs plays a crucial role in enhancing therapeutic outcomes. By conjugating antibodies that specifically bind to cancer cell surface antigens, CNTs can facilitate the targeted delivery of cytotoxic drugs directly to tumor cells, minimizing systemic toxicity. For example, dual-targeting strategies using CNTs have been developed to improve the delivery of neratinib in breast cancer, demonstrating enhanced therapeutic efficacy compared to conventional delivery methods [46]. This targeted approach is particularly beneficial in treating aggressive cancer types, such as triple-negative breast cancer, where conventional therapies often fall short [114]. The ability to deliver drugs directly to cancer cells while sparing healthy tissues represents a significant advancement in cancer treatment. By enhancing the specificity of drug delivery and enabling sensitive biomarker detection, this strategy holds great promise for advancing personalized cancer therapies and improving diagnostic accuracy.

#### 3.2.3. Proteins Functionalized CNTs

Recent studies have shown that functionalizing CNTs with proteins can facilitate targeted drug delivery and improve the detection of cancer biomarkers. For instance, the conjugation of CNTs with tumor suppressor proteins, such as PTEN, has been demonstrated to enhance antitumor activity in breast cancer cells, promoting apoptosis and inhibiting cell proliferation [27]. This targeted approach allows for a more effective treatment strategy, particularly in aggressive cancer types where conventional therapies may be less effective. By leveraging the unique properties of CNTs, researchers are able to create more efficient drug delivery systems that can directly interact with cancer cells, thereby improving therapeutic outcomes.

The protein attachment to CNTs plays a crucial role in enhancing the biocompatibility and stability of these nanomaterials in biological environments. The formation of a protein corona around CNTs can significantly influence their biological interactions and cellular uptake, which is essential for effective drug delivery [115]. For example, studies have indicated that the interaction between CNTs and specific proteins can enhance the targeting of cancer cells, allowing for the selective delivery of chemotherapeutic agents to tumors while minimizing systemic toxicity [116], which is particularly relevant in the treatment of cancers such as colorectal and prostate cancer.

Overall, the attachment of proteins to CNTs offers a promising strategy for advancing cancer therapies and diagnostics. By enhancing drug delivery systems and enabling sensitive biomarker detection, this approach holds great potential for improving personalized cancer treatments and diagnostic accuracy. As research continues to evolve, the applications of protein-functionalized CNTs in oncology are likely to expand, paving the way for innovative solutions in cancer care [68,117].

#### 3.2.4. Carbohydrate Attachment

Recent studies have demonstrated that functionalizing CNTs with carbohydrates, such as hyaluronic acid and mannose, can improve the specificity of drug delivery systems. Hyaluronic acid-modified CNTs have been shown to effectively target specific cancer cell receptors, which are promoting apoptosis in various cancer cells, as a targeted approach, including breast and lung cancers [48]. The incorporation of carbohydrates on CNT surfaces can facilitate the specific binding of cancer biomarkers, allowing for sensitive detection of cancerous cells. For example, studies have shown that glucose-functionalized CNTs can be employed to detect specific glycoproteins linked to colorectal cancer, allowing for early diagnosis and monitoring of treatment responses [66,118]. The high surface area and conductivity of CNTs, combined with the specificity provided by carbohydrate attachments, make these biosensors highly effective for cancer biomarker detection, which is crucial for timely intervention.

In combination with chemotherapeutic agents, carbohydrate-functionalized CNTs have shown promise in enhancing therapeutic outcomes. Research indicates that attaching carbohydrates to CNTs can improve the solubility and stability of anticancer drugs, such as doxorubicin, leading to enhanced drug loading and controlled release profiles [118]. This is particularly beneficial in treating aggressive cancer types, such as triple-negative breast cancer, where conventional therapies often face challenges due to drug resistance [119]. As research continues to evolve, the applications of carbohydrate-functionalized CNTs in oncology are likely to expand, paving the way for innovative solutions in cancer care [19,115].

#### 3.2.5. RNA Attachment

RNA-functionalized CNTs can serve as effective carriers for targeted drug delivery, particularly in the treatment of various cancers such as breast and lung cancer. The conjugation of small interfering RNA (siRNA) to CNTs has been shown to facilitate the targeted silencing of oncogenes, leading to reduced tumor growth and improved therapeutic outcomes [120]. This targeted approach not only enhances the specificity of drug delivery but also minimizes off-target effects, making it a valuable tool in personalized cancer therapy. Moreover, RNA attachment to CNTs has been utilized in the development of advanced biosensors for cancer diagnostics. The incorporation of RNA molecules on CNT surfaces can facilitate the specific binding of cancer-related biomarkers, enabling sensitive detection of cancerous cells. For example, studies have demonstrated that RNA aptamers can effectively capture specific tumor markers, such as prostate-specific antigen (PSA) for prostate cancer detection [121]. Incorporating these aptamers in CNTs is expected to enhance early diagnosis and monitoring of treatment responses.

Research indicates that attaching RNA molecules to CNTs can improve the solubility and stability of therapeutic agents, leading to enhanced drug loading and controlled release profiles [122]. This is particularly beneficial in treating aggressive cancer types, such as triple-negative breast cancer, where conventional therapies often face challenges due to drug resistance [123]. Overall, the attachment of RNA to carbon nanotubes represents a multifaceted approach to advancing cancer diagnostics and treatment. By enhancing drug delivery systems, enabling sensitive biomarker detection, and improving the therapeutic efficacy of anticancer agents, this strategy holds great potential for improving patient outcomes in various cancer types, including breast, lung, and prostate cancers. As research continues to evolve, the applications of RNA-functionalized CNTs in oncology are likely to expand, paving the way for innovative solutions in cancer care [118,124]. Table 1 summarizes the different types of CNTs’ functionality and their biomedical applications.

**Table 1:** Summary of the type of functionalization and example applications.

Type of Functionalization	Functionality Method	Example	Benefit	Application	Reference
**Covalent Functionalization**	Fluorination	Introduction of fluorine atoms to CNTs	Improves dispersion; enhances electronic properties; and increases reactivity	Drug delivery, photothermal therapy	[51,53,56]
	Hydrogenation	Attachment of hydrogen atoms	Modifies electrical conductivity and improves catalytic activity. Works with cisplatin for anticancer properties.	Drug delivery, fuel cells	[19,58,59]
	1,3-Dipolar cycloadditions	Reaction with azomethine ylides	Enhances bioconjugation and allows precise functionalization	Cancer treatment, photodynamic therapy	[62,66]
	Reduction of aryldiazonium salts	Arylation via diazonium salt chemistry	Enables attachment of functional groups for improved targeting	Drug delivery, biosensors	[24,72]
	Reactive species functionalization	Functionalization using reactive oxygen species	Enhances surface reactivity and interactions with biomolecules	Sensors, catalysis, medical applications	[30]
	Organometallic ions	Coordination with transition metals	Improves electronic and catalytic properties	Biosensors, imaging	[81,85,86]
	Carboxylation and further derivation	Carboxyl (-COOH) groups on CNTs	Enhances solubility; allows further modifications	Drug delivery, biosensors	[30,90]
	Amidation and esterification of carboxylic groups	Reaction of carboxylated CNTs with amines/esters	Improves biocompatibility and drug-loading efficiency	Drug delivery, biosensors	[90,91]
	Ionic functionalization of carboxylic groups	Functionalization with ionic groups	Enhances dispersibility in aqueous solutions; improves targeting	Drug delivery, diagnostics	[90]
	Attachment of metallic nanoparticles	Deposition of Au, Ag, and Pt nanoparticles	Improves imaging contrast; enhances targeted therapy	Imaging, cancer treatment	[47,94]
**Non-Covalent Functionalization**	Surfactant functionalization	Use of anionic (SDS, NaDDBS), cationic (CTAB, MATMAC), or non-ionic (Triton X-100) surfactants	Enhances dispersibility and stability in biological systems	Drug delivery, antimicrobial applications	[75]
	Polymer wrapping	Wrapping CNTs with conjugated polymers	Stabilizes CNTs in biological environments; improves targeting	Drug delivery, biosensors	[102]
	Polymer encapsulation	Encapsulation in biocompatible polymers	Improves biocompatibility; enables controlled drug release	Drug delivery, regenerative medicine	[109]
	Polymer absorption	Adsorption of functional polymers onto CNTs	Enhances stability; provides controlled release properties	Drug delivery, biosensors	[112]
	RNA attachment	Conjugation of RNA to CNTs	Enables gene silencing; improves targeted drug delivery	Cancer therapy, genetic engineering	[120,122]
	Protein attachment	Functionalization of CNTs with proteins	Enhances biocompatibility; enables targeted therapy	Cancer treatment, biosensors	[27]
	Antibodies attachment	Conjugation of antibodies to CNTs	Enables specific targeting of cancer cells; enhances biosensor performance	Cancer detection, immunotherapy	[105]
	Carbohydrate attachment	Functionalization with sugars like hyaluronic acid	Improves cellular recognition; enables targeted drug delivery	Cancer therapy, biosensors	[48]

## 4. Carbon Nanotubes in Cancer Diagnosis

### 4.1. Overview

Carbon nanotubes (CNTs) have become a key component in cancer diagnosis due to their unique physical and chemical properties. Their high surface area, electrical conductivity, and mechanical strength make them well-suited for biomedical applications, particularly in detecting cancer biomarkers. Integrating CNTs into diagnostic tools has significantly improved early cancer detection, allowing for more sensitive and accurate identification of malignant cells compared to traditional methods [125,126,127,128].

Beyond detection, CNTs offer versatility through functionalization, which enhances their biocompatibility and targeting abilities. By attaching targeting ligands or drugs, CNTs can selectively bind to cancer cells, improving the accuracy of diagnostic assays [127,129,130,131]. This targeted approach not only aids in diagnosis but also enables therapeutic applications, as CNTs can deliver chemotherapeutic agents directly to tumors, reducing systemic toxicity [132,133]. Their dual role in diagnosis and treatment makes CNTs a crucial tool in advancing nanomedicine.

In addition to biosensing, CNTs are being explored for imaging applications. Their unique optical properties allow them to act as contrast agents in imaging techniques such as fluorescence imaging and magnetic resonance imaging (MRI). Incorporating CNTs into these methods enhances tumor visibility, making it easier to monitor cancer progression and treatment effects [134]. Furthermore, combining CNTs with MRI and computed tomography (CT) has shown potential for real-time tumor tracking and response assessment [135,136]. With their ability to improve both detection and imaging while also offering therapeutic potential, CNTs are shaping the future of cancer diagnostics and treatment, bringing precision and efficiency to modern medicine.

**Figure 4:**
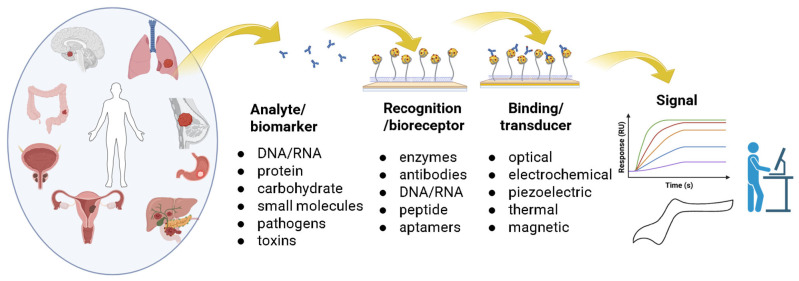
Biosensor components and biosensing processes. Created with BioRender.com.

A biosensor is a device used for analysis that combines a biological recognition component with a transducer, allowing it to detect and measure specific biological substances in different types of samples. It transduces biological responses into a detectable and measurable electrical signal (Figure 4).

CNT-based biosensors can be categorized into various types, such as electrochemical sensors, optical sensors, field-effect transistor (FET) sensors, and hybrid biosensors. Electrochemical biosensors incorporating CNTs have demonstrated exceptional sensitivity and specificity, largely due to CNTs’ high surface area, which facilitates functionality and efficient immobilization of biomolecules [137,138]. These sensors detect target analytes by detecting variations in current or potential upon binding to immobilized biomolecules. The presence of CNTs in the sensor matrix enhances electron transfer kinetics, significantly improving detection limits [137,138,139].

Optical biosensors utilize CNTs’ unique optical properties, such as their ability to quench fluorescence via Förster resonance energy transfer (FRET). This phenomenon has been exploited to create highly sensitive fluorescence-based biosensors capable of detecting low concentrations of biomolecules [140,141]. Additionally, non-covalent functionalization of CNTs with biopolymers and surfactants improves their dispersion in aqueous solutions, further enhancing their performance in optical sensing applications) [141,142].

Field-effect transistor (FET) biosensors based on CNTs offer another promising approach for biosensing. These devices rely on CNTs’ electrical properties to detect changes in the charge state of the sensor surface upon target analyte binding. The high sensitivity of CNT-FETs is attributed to the pronounced change in CNT conductivity in response to biomolecule adsorption. Furthermore, integrating CNTs into flexible and miniaturized sensor platforms has opened new possibilities for point-of-care diagnostics and wearable biosensing devices [142,143].

Beyond individual sensor types, CNTs are also used in hybrid biosensing systems, where they are combined with other nanomaterials, such as graphene and metal nanoparticles, to develop multifunctional sensors. These hybrid systems leverage the complementary properties of different nanomaterials to enhance overall sensing performance [144,145,146,147]. For example, incorporating CNTs with gold and silver nanoparticles has been shown to improve the sensitivity and selectivity of electrochemical biosensors for detecting specific biomolecules [144,145,146,147,148].

### 4.2. CNT-Biosensors for Selective Cancer Detection

Recent advancements in carbon nanotube (CNT)-based biosensors have shown significant promise in the detection of cancer biomarkers. This review categorizes the applications of CNT-biosensors into four main types: electrochemical sensors, optical sensors, field-effect transistor (FET) sensors, and hybrid biosensors. Each category is supported by recent primary research papers that highlight specific CNT-based biosensors, the cancer biomarkers detected, and detailed experimental results.

#### 4.2.1. Electrochemical Biosensors

**Figure 5:**
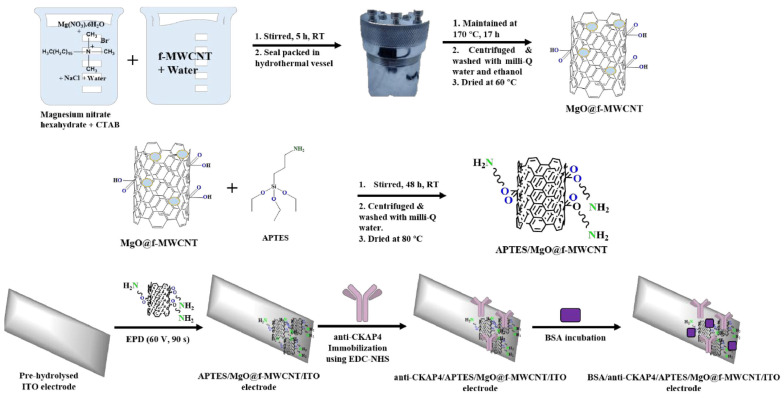
Fabrication process of the working electrode in an electrochemical biosensor for the detection of the lung cancer biomarker CKAP4 (Reused from Shanker et al. [152]).

Electrochemical biosensors have been widely used in the detection of various cancer biomarkers [149,150,151], especially CNT-based electrochemical biosensors [152]. Kumar and co-workers developed a highly sensitive electrochemical immunosensor using a nanoengineered material of magnesium oxide (MgO) on functionalized MWCNTs (f-MWCNT), further modified with APTES, for the early diagnosis of lung cancer biomarker CKAP4. This biosensor achieved a low limit of detection of 6.25 pg/mL. The incorporation of f-MWCNTs enhanced electrical conductivity and provided a large surface area, which improved the biosensor’s sensitivity compared to other metal oxide-based sensors [152]. Figure 5 shows the fabrication methods for the working electrode in electrochemical biosensors.

**Figure 6:**
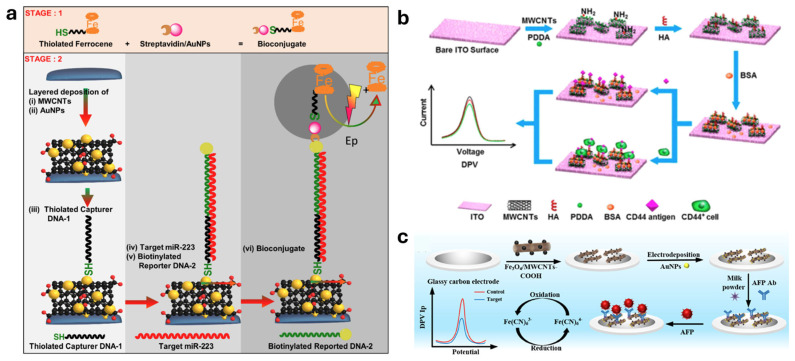
Schematic diagram illustrating the synthesis of: (**a**) a sandwich-type gold/MWCNT nanocomposite–based electrochemical biosensor for non-small cell lung cancer (NSCLC) miR-223 biomarkers (Ref. Bhattacharjee et al. [153]); (**b**) a label-free electrochemical sensor targeting CD44, based on ligand–protein interactions, designed to detect CD44 in human serum and cancer cells (Ref. Zhang et al. [154]; and (**c**) an electrochemical immunosensor for AFP utilizing a modified electrode surface composed of Fe_3_O_4_ nanoparticles, MWCNTs-COOH, and gold nanoparticles (AuNPs) for enhanced sensitivity and performance (Ref. Wu et al. [158]).

Bhattacharjee et al. developed an electrochemical biosensor by employing a gold nanoparticle (AuNPs) functionalized MWCNT nanocomposite for the detection of the non-small cell lung cancer (NSCLC) biomarker miR-223. The MWCNTs enhance the surface area and facilitate electron transfer, while AuNPs further improve conductivity, contributing to the sensor’s high sensitivity. This design, along with a sandwich hybridization assay and a ferrocene-labeled bioconjugate for signal amplification, enables a low detection limit of 0.73 pM and good selectivity for miR-223 over homologous sequences (Figure 6a) [153].

In another study conducted by Zhang et al., a label-free electrochemical sensor was developed by utilizing MWCNTs for the detection of CD44, a cell surface glycoprotein that is overexpressed in various cancers, including lung and breast cancers [154]. The fabricated sensor was constructed on an indium tin oxide (ITO) electrode modified with MWCNTs and polydiallyldimethylammonium (PDDA) to enhance the electrochemical performance. The sensor showed a linear response to CD44-positive cancer cells, indicating its reliability for clinical applications. The electrochemical response was significantly enhanced due to the high surface area and conductivity of the MWCNTs, which facilitated the binding of CD44. The detection was based on the electrochemical impedance spectroscopy (EIS) technique, which allowed for real-time monitoring of the binding events between the sensor and the cancer cells (Figure 6b) [154].

Electrochemical biosensing approaches were also explored for the detection of biomarkers for cancer [155,156,157]. Yang and co-workers reported an electrochemical immunosensor utilizing MWCNTs-COOH functionalized with Fe_3_O_4_ and AuNPs for the detection of the liver cancer marker alpha-fetoprotein (AFP). The use of functionalized MWCNTs-COOH provides a large surface area for stable antibody immobilization through covalent bonding, enhancing the electrochemical signal and contributing to high sensitivity and selectivity for AFP. The developed sensor achieved a low detection limit of 1.09034 pg/mL [158]. The fabrication of the functionalized MWCNTs-COOH working electrode for the electrochemical sensors is illustrated in Figure 6c.

Xu et al. investigated a CNT-based electrochemical sensor for the detection of alpha-fetoprotein (AFP), a well-known biomarker for hepatocellular carcinoma (liver cancer) [159]. The sensor utilized a composite of conducting polymers and CNTs, which provided a highly effective electrochemical sensing material. The study reported a detection limit of 0.20 μM for AFP, which is significant for early diagnosis and monitoring of liver cancer. The electrochemical detection was performed using cyclic voltammetry (CV), which allowed for the assessment of the electrochemical behavior of the AFP biomarker upon binding to the sensor surface [159]. Makableh et al. developed an electrochemical Aptasensor for the early detection of HER2, a biomarker for breast cancer. The biosensor utilizes a screen-printed carbon electrode modified with gold nanoparticles (GNPs) and MWCNTs to enhance response and selectivity. Employing electrochemical techniques like differential pulse voltammetry (DPV), the Aptasensor achieved a very low detection limit of 4.4 Pg/mL with a response time of approximately 5 min [160].

#### 4.2.2. Field-Effect Transistor (FET) Sensors

**Figure 7:**
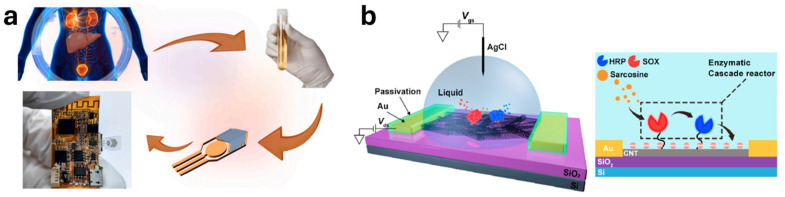
Schematic diagram illustrating FET immunosensors: (**a**) a non-invasive approach for detecting exosomal microRNA-122 in the urine of liver cancer patients using MC@CNT-iFETs (Ref. Zhang et al. [163]); and (**b**) a CNT-FET modified with enzymatic cascade reactors (Ref. Liu et al. [167]).

Field-Effect Transistor (FET) Sensors based on functionalized CNTs have been explored for the detection of biomarkers for diverse cancers, including liver, breast, prostate, and lung cancer [161,162,163]. Recently, You and co-workers developed a portable field-effect transistor (FET) biosensor utilizing a novel semiconductor material composed of metal carbide@carbon nanotubes (MC@CNTs) for the non-invasive detection of exosomal microRNA-122, a biomarker for liver cancer, in urine. The incorporation of CNTs enhances the electrical conductivity and transconductance of the metal carbide, contributing to significantly improved sensitivity. This MC@CNT-iFETs device achieved an ultralow detection limit of 0.12 fM for microRNA-122, and its high specificity is attributed to the use of a specific aptamer for microRNA-122 recognition (Figure 7a) [163].

Choi and co-workers reported a method for the real-time and highly sensitive monitoring of adenosine receptor activities in non-small cell lung cancer (NSCLC) cells. The method utilizes an electrochemical transduction mechanism via a carbon nanotube field-effect transistor (CNT-FET) hybridized with NSCLC cells. The CNT-FET is functionalized with a fibronectin coating for stable immobilization of the cancer cells expressing adenosine receptors. This platform enables the detection of adenosine, a key molecule in lung cancer biology, with an ultralow detection limit of 1 fM [162].

In another study, a CNT FET biosensor was developed for the detection of carcinoembryonic antigen (CEA), a well-known biomarker for lung cancer. The biosensor was functionalized with specific antibodies against CEA and operated using electrostatic coupling to detect biomolecular interactions. The results indicated a wide detection range from 1 fg/mL to 1 ng/mL and sensitivity of 72 ag/mL, demonstrating its effectiveness in clinical applications for lung cancer diagnostics [164].

Liu et al. developed a semiconductor carbon nanotube (CNT)-based field-effect transistor (FET) DNA sensor designed for the detection of the BRCA1 gene, a key biomarker associated with breast cancer. The CNT-FET, featuring a floating gate and high κ dielectric, was optimally functionalized with peptide nucleic acid (PNA) probes. It achieved excellent sensitivity with a low detection limit of 1.38 aM and successfully distinguished between patients and healthy individuals [165].

Li et al. presented a CNT field-effect transistor (FET) biosensor specifically designed for the ultrasensitive and label-free detection of exosomal miRNA21, a biomarker associated with breast cancer. The biosensor exhibited a detection limit of 0.87 aM, showcasing its potential for early diagnosis. The study highlighted the ability of the CNT FET to provide real-time monitoring of biomarker levels, which is crucial for timely intervention in breast cancer treatment [166].

Wei and colleagues recently developed a high-purity semiconductor carbon nanotube-based field-effect transistor (CNT-FET) integrated with enzymatic cascade reactors, enabling highly sensitive detection of sarcosine, a biomarker associated with biopsy-positive prostate cancer. The CNT network improves reaction efficiency, enhancing signal transduction, leading to a limit of detection as low as 105 zM. This CNT-FET sensor demonstrated the ability to precisely distinguish prostate cancer samples from benign prostatic hyperplasia samples with higher accuracy than conventional PSA testing (Figure 7b) [167].

#### 4.2.3. Optical Sensors

Several types of optical biosensors have been developed and used for the early detection of cancer biomarkers, including photonic crystals, surface plasmon resonance (SPR) spectroscopy [168,169], surface-enhanced Raman spectroscopy (SERS), and colorimetric sensors [170,171]. Due to the advantages of the localized surface plasmon resonance (LSPR) phenomenon, optical SPR biosensors for biomolecule and cancer detection have been extensively studied [155,156,157,172]. However, plasmonic nanoparticles (NPs) are commonly used to enhance electrochemical detection with CNT-SPR biosensors, and there are limited recent studies investigating SPR optical detection of CNT-SPR optical biosensors. Recently, Karki et al. proposed a surface plasmon resonance (SPR) sensor incorporating carbon nanotubes (CNTs) for the ultra-sensitive detection of colorectal cancer. The sensor, utilizing a CaF_2_ prism/Cu/CNT/HfSe_2_ multilayer structure, detects changes in the refractive index between healthy and cancerous colorectal tissue via shifts in the resonance angle, leveraging the optical property of SPR. The inclusion of a 1 nm thick CNT layer enhances the sensor’s sensitivity, achieving a maximum sensitivity of 387.60 deg/RIU specifically for colorectal cancer detection. This design presents a promising approach for early and less invasive colorectal cancer detection [157]. The study by Uniyal et al. proposes a surface plasmon resonance (SPR) sensor utilizing fluorinated graphene and carbon nanotubes (CNTs) for the detection of SARS-CoV-2 viral particles. This work highlights the diagnostic potential of this sensor configuration, with the Introduction mentioning the vital role of diagnostic testing in outbreak containment. The use of fluorinated graphene and CNTs is explored to enhance the sensitivity of the SPR sensor for this diagnostic application [57]. The study also calculates the Limit of Detection (LOD) for the considered SARS-CoV-2 concentrations. For the suggested sensor configuration with one layer of fluorinated graphene and five layers of CNTs, the maximum LOD attained is 3.07 × 10^−6^ RIU for a 125 nM concentration of coronavirus [57].

An optical biosensor for Chronic Lymphocytic Leukemia (CLL) biomarker detection using MWCNTs has been developed by Kala and co-workers. The MWCNTs are functionalized with silane to enable antibody immobilization. The sensing mechanism relies on monitoring Fermi level fluctuations induced by the binding of CLL cells, detected through photocurrent and photoconductive response using a 635 nm laser. The developed biosensor achieved a limit of detection of 27 cells/mL [173].

CNT-based fluorescence sensors are widely used [174], with CNTs also serving as effective fluorescence quenchers. Bio-recognition elements, such as oligonucleotides, can be fluorescently labeled and non-covalently attached to CNTs, where their fluorescence is quenched via Förster resonance energy transfer (FRET). In the presence of analyte molecules, these oligonucleotides preferentially bind to the analyte, displacing the CNTs and leading to a significant fluorescence increase [175].

Williams et al. utilized fluorescent SWCNTs as optical transducers for in vivo nanobiosensors. These SWCNTs are often combined with materials like ssDNA, antibodies, peptides, or polymers for dispersion and specific biomarker recognition. While various biomarkers are detected, including the ovarian cancer biomarker HE4 and surface proteins on prostate cancer cells (PSMA), and pH change detection is demonstrated in ovarian cancer xenografts, this review specifies different detection levels for various analytes rather than a singular limit for a specific cancer type. For instance, the HE4 sensor could discriminate between mice injected with 10 pmol of HE4 and controls [176].

Table 2 summarizes the types of functionalized CNTs that have been explored for the detection of diverse types of cancers and their biomarkers. Recent primary research on CNT-based biosensors for cancer detection has revealed significant advancements across various sensor types. Electrochemical and FET sensors have demonstrated high sensitivity and specificity for cancer biomarkers, making them promising candidates for clinical applications. Additionally, while optical biosensors have been extensively explored for cancer biomarker detection, research on CNT-based optical sensors, particularly fluorescence-based sensors, has primarily focused on their applications in cancer imaging rather than biomarker detection.

**Table 2:** Summary of the types of biosensors and example applications.

Type of Biosensor	Type of CNT Used	Type of Cancer/Biomarker/Molecule Detected	Type of Recorded Signal	Detection Limit	Ref.
**Electrochemical**	Functionalized MWCNT (f-MWCNT)	Lung cancer/CKAP4	Change in current or potential (implied)	6.25 pg/mL	[152]
	Gold nanoparticle (AuNPs) functionalized MWCNT	Non-small cell lung cancer (NSCLC)/miR-223	Change in current or potential (implied)	0.73 pM	[153]
	MWCNTs	Lung and breast cancers/CD44 (cancer cells)	Electrochemical impedance spectroscopy (EIS)	5.94 pg/mL	[154]
	MWCNTs-COOH functionalized with Fe_3_O_4_ and AuNPs	Liver cancer/alpha-fetoprotein (AFP)	Electrochemical signal	1.09034 pg/mL	[158]
	CNTs combined with conducting polymer	Liver cancer/alpha-fetoprotein (AFP)	Cyclic voltammetry (CV)	0.20 μM	[159]
	MWCNTs modified with gold nanoparticles (GNPs)	Breast cancer/HER2	Differential pulse voltammetry (DPV)	4.4 Pg/mL	[160]
**Field-Effect Transistor** (**FET**)	Metal carbide@carbon nanotubes (MC@CNTs)	Liver cancer/exosomal microRNA-122	Change in electrical conductivity/transconductance	0.12 fM	[163]
	CNT	Non-small cell lung cancer (NSCLC) cells/adenosine receptor activity/adenosine	Electrochemical transduction (implied through hybridization with CNT-FET)	1 fM	[162]
	CNT functionalized with specific antibodies	Lung cancer/carcinoembryonic antigen (CEA)	Change in electrical conductivity (implied)	72 ag/mL	[164]
	Semiconductor CNT functionalized with peptide nucleic acid (PNA) probes	Breast cancer/BRCA1 gene	Change in electrical conductivity (implied)	1.38 aM	[165]
	CNT-FET coated with fibronectin	Breast cancer/exosomal miRNA21	Change in electrical conductivity (implied)	0.87 aM	[166]
	High-purity semiconductor CNT modified with enzymatic cascade reactors	Prostate cancer/sarcosine	Enhanced signal transduction (change in electrical conductivity implied)	105 zM	[167]
**Optical**	CNTs in a CaF_2_ prism/Cu/CNT/HfSe2 multilayer structure	Colorectal cancer/refractive index difference between healthy and cancerous tissue	Shift in resonance angle	Maximum sensitivity of 387.60 deg/RIU (for colorectal cancer detection)	[157]
	MWCNTs functionalized with silane	Chronic Lymphocytic Leukemia (CLL)/CLL cells	Photocurrent and photoconductive response	Limit of detection of 27 cells/mL	[173]
	SWCNTs (as quenchers)	Ovarian cancer/HE4; prostate cancer/PSMA (among others not specific to one cancer in this description)	Fluorescence increases upon analyte binding	For HE4: could discriminate between mice injected with 10 pmol and controls	[176]

### 4.3. CNTs in Cancer Imaging Modalities

Recent advancements in CNT-based biosensors have demonstrated significant potential in enhancing cancer imaging modalities [177,178,179]. CNTs have gained significant attention in the field of fluorescence imaging due to their unique optical properties, including strong fluorescence, low photobleaching, and tunable emission wavelengths. Recent studies have explored various applications of CNTs in fluorescence imaging, particularly in biological systems for cancer detection and monitoring [174]. This section reviews recent primary research papers that highlight the advancements in CNT-based fluorescence imaging.

#### 4.3.1. CNTs in Fluorescence Imaging

Hendler-Neumark et al. investigated the use of SWCNTs as fluorescent probes for in vivo imaging within the gastrointestinal tract of *Caenorhabditis elegans* (*C. elegans*) nematodes. The study demonstrated that DNA-functionalized SWCNTs exhibit remarkable photostability compared to traditional organic fluorescent dyes, allowing for prolonged imaging of dynamic biological processes without significant photobleaching. The researchers reported that SWCNTs enabled real-time imaging of cellular events over extended periods, showcasing their potential for long-term tracking of biological phenomena) [180]. This property is particularly advantageous in cancer research, where continuous monitoring of tumor dynamics is crucial.

In their study, Zhou et al. enhanced imaging capabilities by creating a nanocomposite, GdN@CQDs-MWCNTs, which combines the deep tissue penetration of magnetic resonance imaging (MRI) with the single-cell sensitivity of fluorescence imaging (FI). The research focused on imaging and treating A549 lung cancer cells in living mice. The main finding was the successful development of a magnetofluorescent nanocomposite capable of targeted drug delivery and synergistic chemo/photothermal therapy, with fluorescence imaging used to track cellular uptake and in vivo distribution [177].

Kojima et al. explored the effects of oxygen-doping on SWCNTs to enhance their fluorescence properties for use as near-infrared labels in immunoassays. The introduction of oxygen atoms into the CNT structure resulted in a red shift in the fluorescence wavelength, improving the contrast in biological imaging applications. The study demonstrated that Streptavidin oxygen-doped SWCNTs could effectively reduce autofluorescence in living cells, thereby increasing the detected signal and enhancing image quality [181]. This functionalization strategy opens new avenues for using CNTs in fluorescence imaging, particularly in complex biological environments where background noise can obscure signals.

**Figure 8:**
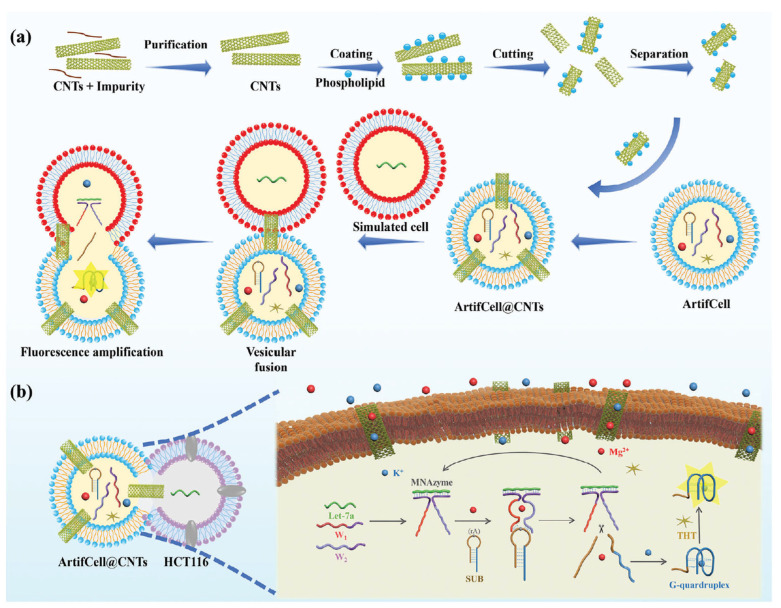
Schematic of the ArtifCell@CNTs-based probe transfer-enhanced sensing strategy. (**a**) Purification of CNTs and the preparation of Artif-Celll@CNTs for fluorescence sensing. (**b**) Illustration of the ArtifCell@CNTs-based sensing specifically targeting let-7a (Ref. Wu et al. [182]).

In a recent study, Wu et al. developed an artificial cell-based sensing system where embedding carbon nanotubes (CNTs) in the artificial cell membranes significantly enhances fluorescence imaging of intracellular biomarkers. These embedded CNTs function as artificial channels, facilitating the faster and more efficient transfer of signal probes, such as nucleic acids and fluorescent dyes, across cell membranes. This enhanced probe transfer, mediated by CNTs, leads to improved fusion between the artificial cells and target cells, allowing the probes to selectively hybridize with intracellular target miRNAs and trigger a fluorescence signal. Consequently, the study demonstrates enhanced fluorescence signal and improved visualization of let-7a miRNA within living colon cancer cells, showcasing the potential of CNTs to overcome limitations in probe delivery for intracellular imaging (Figure 8) [182].

**Figure 9:**
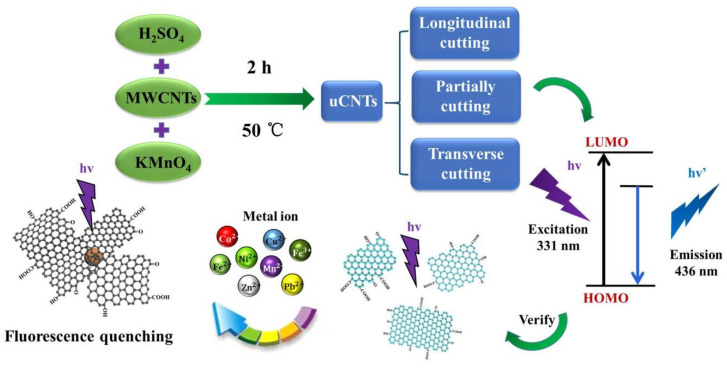
Illustration of the unzipping process of a carbon nanotube (CNT) into a graphene nanoribbon, along with the corresponding energy profile (Ref. Chen et al. [183]).

Chen et al. investigated the photoluminescence properties of unzipped MWCNTs (uMWCNTs) and their potential applications in fluorescence imaging. The study reported that uMWCNTs exhibited strong fluorescence with a peak emission of around 550 nm, which is suitable for various imaging applications. The authors noted that the unique electronic properties of CNTs allow for efficient charge separation and migration, which enhances their fluorescence intensity (Figure 9) [183]. This property can be leveraged to improve the sensitivity of fluorescence imaging techniques in detecting cancer biomarkers.

Mandal et al. demonstrated the use of sp^3^ defect-tailored CNTs for near-infrared (NIR-II) single-particle imaging in live brain slices. The study achieved high signal-to-noise ratios using low excitation intensities, which is crucial for minimizing photodamage in sensitive biological tissues. This work highlights the potential of CNTs as single-molecule probes for advanced imaging techniques, paving the way for their application in real-time monitoring of tumor microenvironments [184]. The study also shows that para-nitroaryl functionalized SWCNTs (f-SWCNTs) exhibit brighter fluorescence in the NIR-II region with significantly lower excitation intensities compared to non-functionalized SWCNTs (unf-SWCNTs). This enhanced performance of f-SWCNTs allows for higher signal-to-noise ratio imaging in live tissue using an order of magnitude less light excitation than required for unf-SWCNTs [184]. The ability to visualize individual CNTs in biological systems could lead to breakthroughs in understanding cancer biology and treatment responses.

The application of carbon nanotubes in fluorescence imaging represents a significant advancement in biomedical imaging technology. Recent studies have demonstrated that CNTs, particularly SWCNTs and MWCNTs, possess unique optical properties that make them suitable for long-term imaging, enhanced contrast, and live tracking of biological functions. The functionalization of CNTs further enhances their imaging capabilities, allowing for targeted applications in cancer diagnostics and therapy. As research continues to evolve, CNTs are willing to play a critical role in advancing fluorescence imaging techniques in oncology and beyond.

#### 4.3.2. CNT-Enhanced MRI

Carbon nanotubes (CNTs) have emerged as promising materials in the field of magnetic resonance imaging (MRI), primarily due to their unique properties that can enhance imaging contrast and provide multifunctional capabilities. Furthermore, Gao et al., in their recent review, highlighted the potential of functionalized CNTs in enhancing MRI capabilities. They discussed how surface modifications could be tailored to optimize the targeting of specific cancer biomarkers, thereby increasing the specificity of MRI imaging [19]. This targeted approach can lead to more accurate tumor detection and characterization, which is vital for effective treatment planning. This section reviews recent studies focusing on the application of CNTs in MRI, detailing the specific types of CNTs used, their functionalization, and the experimental results related to their effectiveness as contrast agents.

One significant study by Mehri-Kakavand et al. investigated the use of gadolinium (Gd)-loaded CNTs as MRI contrast agents. The researchers synthesized Gd_3+_@CNTs-PEG and compared their performance with Gadovist^®^, a commercially available contrast agent. The results indicated that Gd_3+_@CNTs-PEG exhibited superior relaxivity, enhancing the proton transverse relaxation times (T2) significantly. The study reported a relaxivity value of 12.5 mM^−1^s^−1^ for the CNT-based contrast agent, compared to 5.5 mM^−1^s^−1^ for Gadovist^®^ [185]. This enhanced relaxivity suggests that CNTs can provide improved imaging quality and sensitivity in MRI applications.

In another study, Wolski et al. designed a multimodal, pH-sensitive drug carrier using CNTs that could be visualized via MRI due to the presence of superparamagnetic nanoparticles. The study demonstrated that these constructs could effectively unload their drug cargo at the tumor site, while also enhancing MRI contrast. The superparamagnetic nanoparticles increased the proton transverse relaxation times, acting as effective MRI contrast agents [186]. This dual functionality of drug delivery and imaging is particularly advantageous in cancer therapy, where monitoring treatment response is crucial.

The functionalization of CNTs plays a critical role in their effectiveness as MRI contrast agents. For instance, Glória et al. explored the solubilization and characterization of MWCNTs for biomedical applications. They found that the adsorption of polymers significantly improved the dispersion of CNTs in biological media, enhancing their compatibility for use in MRI [187]. This improved solubilization is essential for ensuring that CNTs can circulate effectively in the bloodstream and reach target tissues.

The application of carbon nanotubes in MRI represents a significant advancement in imaging technology. Their unique properties, including enhanced relaxivity and the ability to be functionalized for targeted delivery, position CNTs as valuable tools in the field of medical imaging. Ongoing research into the optimization of CNTs for MRI will likely yield further improvements in imaging quality and specificity, ultimately benefiting patient care in oncology.

#### 4.3.3. CNTs in Computed Tomography (CT)

In the field of computed tomography (CT), carbon nanotubes (CNTs) have attracted a lot of interest because of their special chemical and physical characteristics that potentially improve imaging capabilities. Recent research has examined a number of CNT applications in CT, with an emphasis on their usage as contrast agents and X-ray sources. This section summarizes the most recent research on CNTs in CT imaging, including particular studies, CNT types, and experimental outcomes [188,189,190,191]. Jo reported a study on optimizing image quality in a carbon nanotube (CNT)-based rectilinear Digital Tomosynthesis System (DTS). This system is explored as an alternative to traditional CT in External Beam Radiation Therapy (EBRT) for cancer treatment, aiming to reduce radiation exposure and acquisition time. The research highlights the importance of balancing image quality and computational efficiency for real-time patient positioning in radiotherapy [192].

Dillon et al. presented a novel hardware configuration using carbon nanotube (CNT) field emission source arrays for fast 3D X-ray imaging in radiotherapy. This technology aims to improve upon current CT imaging limitations, particularly for cancers affected by respiratory motion. Their simulation study uses a 4D patient phantom with an implanted tumor moving due to respiration, suggesting a focus on lung cancer imaging. The goal is to enable volumetric imaging before and during treatment delivery [193].

One of the most promising applications of CNTs in CT is their use as X-ray sources. Kim et al. developed a carbon nanotube emitter X-ray source aimed at improving the resolution of micro-computed tomography. Their research demonstrated that CNT-based X-ray sources could significantly reduce radiation exposure while maintaining high resolution. The study reported that the CNT emitter achieved a spatial resolution of 2.5 μm, which is a substantial improvement over traditional X-ray sources [189]. This advancement is crucial for applications requiring detailed imaging, such as in oncology, where precise tumor localization is essential [194]. In another study, Kim et al. further explored the development of a high-speed micro-computed tomography system based on a CNT emitter X-ray source. This system was designed to enhance imaging speed while maintaining image quality. The results indicated that the CNT-based system could achieve imaging speeds of up to 30 frames per second, which is significantly faster than conventional systems [191]. The ability to capture high-speed images is particularly beneficial in dynamic imaging scenarios, such as monitoring tumor response to treatment.

Moreover, Zulkifli et al. examined the effects of CNTs on the flexural properties of carbon fiber/epoxy composites, indirectly suggesting that CNTs could enhance the mechanical properties of materials used in CT imaging systems [195]. While this study did not focus directly on CT imaging, it underscores the potential of CNTs to improve the structural integrity of imaging devices, which could lead to better imaging outcomes.

The application of carbon nanotubes in acoustic imaging represents a significant advancement in the field of cancer diagnostics. Studies have revealed that CNT-based ultrasound contrast agents can enhance imaging sensitivity and specificity, providing valuable tools for early cancer detection. As research continues to evolve, the potential for CNTs to revolutionize ultrasound imaging in oncology remains substantial.

## 5. Conclusions

Functionalized CNTs hold significant promise as versatile tools for advancing cancer diagnosis and imaging. The urgent global need for effective early cancer detection, highlighted by high incidence and mortality rates and substantial economic costs, underscores the importance of innovative diagnostic approaches. While advances in cancer treatment exist, early detection and imaging remain critical challenges that CNTs are poised to address.

The unique structural properties of CNTs, coupled with the ability to tailor their characteristics through various functionalization strategies (both covalent and non-covalent), enable enhanced biocompatibility, selectivity, and performance in biomedical applications. This review has explored the application of functionalized CNTs in diverse diagnostic modalities, including highly sensitive biosensors such as electrochemical, optical, and field-effect transistor (FET) sensors capable of detecting a wide range of cancer biomarkers with impressive detection limits across various cancer types. Furthermore, the utility of CNTs extends to enhancing cancer imaging techniques such as fluorescence imaging, magnetic resonance imaging (MRI), computed tomography (CT), and ultrasound imaging, often improving contrast, resolution, and targeting capabilities.

Despite these advancements, there is a continued need for the development of improved CNT-based diagnostic platforms and strategies to overcome the challenges associated with their widespread adoption in medical practice. Further research should focus on optimizing functionalization techniques, enhancing biocompatibility and long-term safety, and translating these promising findings into clinically viable tools for earlier and more accurate cancer diagnosis. Future direction in this field includes utilizing CNTs as carriers for testing cancer biomarkers and this necessitates robust diagnostic clinical trials to explore their full potential as safe, effective, and accurate diagnostic tools in Cancer diagnostics. The continued exploration of novel functionalization methods and their integration into diverse diagnostic and imaging modalities will undoubtedly pave the way for significant progress in mitigating the consequences of this global health concern.

## Data Availability

No new data were created or analyzed in this study. Data sharing is not applicable to this article.
